# Folate promotes *S*-adenosyl methionine reactions and the microbial methylation cycle and boosts ruminants production and reproduction

**DOI:** 10.1186/s13568-018-0592-5

**Published:** 2018-04-23

**Authors:** Imtiaz Hussain Raja Abbasi, Farzana Abbasi, Lamei Wang, Mohamed E. Abd El Hack, Ayman A. Swelum, Ren Hao, Junhu Yao, Yangchun Cao

**Affiliations:** 1 Department of Animal Nutrition and Feed Science, College of Animal Science and Technology, Northwest A&F University, Yangling, 712100 Shaanxi People’s Republic of China; 2 School of Life Science and Engineering, Southwest University of Science and Technology, Mianyang, 621010 Sichuan People’s Republic of China; 3 Department of Poultry, Faculty of Agriculture, Zagazig University, Zagazig, 44511 Egypt; 4 Department of Animal Production, College of Food and Agriculture Sciences, King Saud University, P.O. Box 2460, Riyadh, 11451 Saudi Arabia

**Keywords:** Epigenetic, DNA stability, Folate, Microbial methylation, Ruminants, Vitamin B_12_

## Abstract

Folate has gained significant attention due to its vital role in biological methylation and epigenetic machinery. Folate, or vitamin (B_9_), is only produced through a de novo mechanism by plants and micro-organisms in the rumen of mature animals. Although limited research has been conducted on folate in ruminants, it has been noted that ruminal synthesis could not maintain folate levels in high yielding dairy animals. Folate has an essential role in one-carbon metabolism and is a strong antiproliferative agent. Folate increases DNA stability, being crucial for DNA synthesis and repair, the methylation cycle, and preventing oxidation of DNA by free radicals. Folate is also critical for cell division, metabolism of proteins, synthesis of purine and pyrimidine, and increasing the de novo delivery of methyl groups and *S*-adenosylmethionine. However, in ruminants, metabolism of B_12_ and B_9_ vitamins are closely connected and utilization of folate by cells is significantly affected by B_12_ vitamin concentration. Supplementation of folate through diet, particularly in early lactation, enhanced metabolic efficiency, lactational performance, and nutritional quality of milk. Impaired absorption, oxidative degradation, or deficient supply of folate in ruminants affects DNA stability, cell division, homocysteine remethylation to methionine, de novo synthesis of *S*-adenosylmethionine, and increases DNA hypomethylation, uracil misincorporation into DNA, chromosomal damage, abnormal cell growth, oxidative species, premature birth, low calf weight, placental tube defects, and decreases production and reproduction of ruminant animals. However, more studies are needed to overcome these problems and reduce enormous dietary supplement waste and impaired absorption of folate in ruminants. This review was aimed to highlight the vital role of folic acid in ruminants performance.

## Introduction

An inappropriate balance of essential nutrients in the diet promotes health disorders and impedes the development of dairy animals. Bacterial populations in the rumen of ruminants synthesize a large amount of B vitamins and these essential nutrients are also supplemented in rations aimed to cover the needs of the animals and prevent deficiency disorder. However, the amounts of B vitamins in these rations are not adequate to achieve the best performance of high-yield dairy animals (Rosenblatt and Fenton 2001; Abbasi et al. 2014; Li et al. 2016). Folate (C_19_H_19_N_7_O_6_; folic acid or vitamin B_9_) has many forms, namely, folic acid (synthetic form) (Berry et al. 2010), methyltetrahydrofolate, folinic acid, methenyltetrahydrofolate, folacin, tetrahydrofolic acid, pteroyl-l-glutamate, and in the body, the liver acts as a major storage place for folates stored in the form of polyglutamates (Darby and jones 1945; Fenech 2012). In recent years, folate has come into focus due to its protective role, its essential role in metabolism, and because it is a key agent in de novo processes and epigenetics. Folate is important for hematopoiesis and function of red, white and new blood cells. Folate is also vital for biochemical functions in mammals including one-carbon methyl transfer reactions, the synthesis of purine, RNA, pyrimidine, and DNA by methylation, preventing changes in the DNA and abnormal cell development (Blount et al. 1997; Choi and Mason 2000; Kronenberg et al. 2008; Figueiredo et al. 2009), gene expression, and neurotransmitter functions (Ghoshal et al. 2006; Pogribny et al. 2008). Furthermore, folate contributes significantly to amino acids synthesis (Fournier et al. 2002; Shinohara et al. 2006), and is an important agent in the formation of the primary methylating agent, *S*-adenosylmethionine (SAM) (Bailey and Gregory 1999; Linhart et al. 2008). Folate plays an especially important role during the cells division cycle, growth, and the early gestation period (Kamen 1997). Ingredients which contain low folate along with an excessive number of unstable forms of this vitamin making analysis of dietary folate very interesting in ruminant animals.

Defective or impaired folate transportation or metabolism, resulting folate shortage and consequent 5-methyltetrahydrofolate depletion occurs (Rosenblatt and Fenton 2001). It was noted that, in most species, ingested folic acid rapidly reduced and methylated across the gastrointestinal wall before appearing as 5-methyl-tetrahydrofolate (5-methyl-THF) in the liver (LeGrusse et al. 1993). This cycle is irrevocable and a methyl group is released during conversion of 5-methyl-THF to homocysteine for use in methionine and tetrahydrofolate (THF) synthesis (Bassler 1997). THF is an active form of folate and functions as an acceptor of one-carbon units from multiple reactions (Xue and Snoswell 1985). However, methionine requirements in ruminants increase during lactation and methionine production is maintained through remethylation. This is because lactation duration increases the need for methylated agents (choline, creatine, creatinine, and carnitine), and methionine is needed to promote milk protein synthesis and production (Abbasi et al. 2017). However, net absorption or methylated agents is low and meeting the need for these methylated compounds requires de novo synthesis (Snoswell and Xue 1987) from gluconeogenic precursors, glycine or serine, the primary sources for the required methyl groups (Armentano 1994). During early lactation, glucose synthesis increases and this may generate a scarcity of resources for the de novo synthesis of methylated precursors. Under such a condition, without additional sources of methyl groups including methionine, which further metabolizes into homocysteine and cysteine, methyl donor deficiency results in poor milk protein or yield performance (Scott 1999; Abbasi et al. 2018). Furthermore, when methionine supply in dairy cow rations is low (NRC 2001), folic acid supplementation promotes better milk performance and proper methylation (Girard et al. 1995; Girard and Matte 1998; Smith et al. 2012). These studies suggest that during parturition, dietary folate requirements increase and folic acid synthesized by rumen microflora from rumen unprotected folate did not fulfill the needs of the animal. Folate deficiency first established in erythrocytes cells and then in bone marrow due to deficiency of RNA and DNA for normal cell division processes and subsequent protein/enzyme synthesis deficiency causing premature birth, low calf weight, and increased placental tube deformation risk (Blom et al. 2006; Gabory et al. 2011). However, folic acid supplementation through rations supplied methionine for anabolic output and enhanced efficiency of one-carbon units transfer (Wallingford et al. 2013). A satisfactory supply of methyl group sources or cofactors (folates, B_12_) promotes the different metabolic pathways and improves milk production. Therefore, the present review was undertaken to asses and elucidates the interactions and vital role of folic acid in ruminants performance.

## Metabolism of folate

Almost all mammalian cells obtain 5-methyl-THF monoglutamate as exogenous folate, the kind of folate most commonly transported in the blood stream. In its natural form, folate occurs as polyglutamate in food ingredients. When this folate is digested, glutamate is removed in the rumen, and a methyl group is added and fascinated via cells. After methyl group activation, folate is available as folate coenzyme for DNA synthesis (Fowler 2001; Liu and Ward 2010). Furthermore, natural dietary folate possesses five to seven glutamate side chains residues linked by g-peptide linkages (Gregory 1996; Wallingford et al. 2013). In most species, dietary folate is absorbed via the small intestine and then moved to the liver, where it is metabolized into 5-methyl-THF by dihydrofolate reductase and then polyglutamated for cellular retention (Stanger 2002; Liu and Ward 2010). Next, THF is converted to 5,10-methylene-THF through the vitamin B_6_ dependent serine hydroxymethyltransferase reaction before being reduced irrevocably into 5-methyl-THF by methylenetetrahydrofolate reductase (MTHFR) enzyme. 5-Methyl-THF works as a co-substrate and a key methyl donor for methionine synthesis from homocysteine. Methionine has a key role and contributes SAM, which has a vital role in 5-methylcytosine forming methylation reactions catalyzed by DNA methyltransferases (DNMTs) (Stanger 2002; Liu and Ward 2010; Crider et al. 2012; Abbasi et al. 2018). In this pathway, key genes include those related to relocating the methyl group to homocysteine, such as those encoding methylenetetrahydrofolate (MTRR), reduced folate carrier (RFC), and vitamin B_12_-dependent methionine synthase (MTR) (Zhang et al. 2013). However, 5-methyl-THF is a poor enzyme for elongating glutamate chains (Shane 1989), and vitamin B_12_ is needed to enable elimination of the methyl group for methionine synthesis (Shane et al. 1977). It has been noted that demethylation of 5-methyl-THF is a limiting reaction for cellular accretion of folates (Lucock 2007). The uptake of 5-methyl-THF by cells is maintained through transporters, namely, proton coupled folate transporter (PCFT) and reduced folate carrier (RFC). The receptors involved are folate receptor alpha (FRa) and folate receptor beta (Frb) (Matherly and Goldman 2003). Alteration or mutation can damage the gene coding PCFT and cause inherited folate malabsorption disorder (Zhao et al. 2007, 2009). Then PCFT plays a major role in intestinal folate absorption; when folate binds to PCFT receptors, it is taken up by epithelial cells through receptor-mediated endocytosis and from there, easily passes into the interstitium and cerebrospinal fluid of the brain (Blount et al. 1997; Wu and Pardridge 1999).

## Folate deficiency and genomic instability

**Fig. 1:**
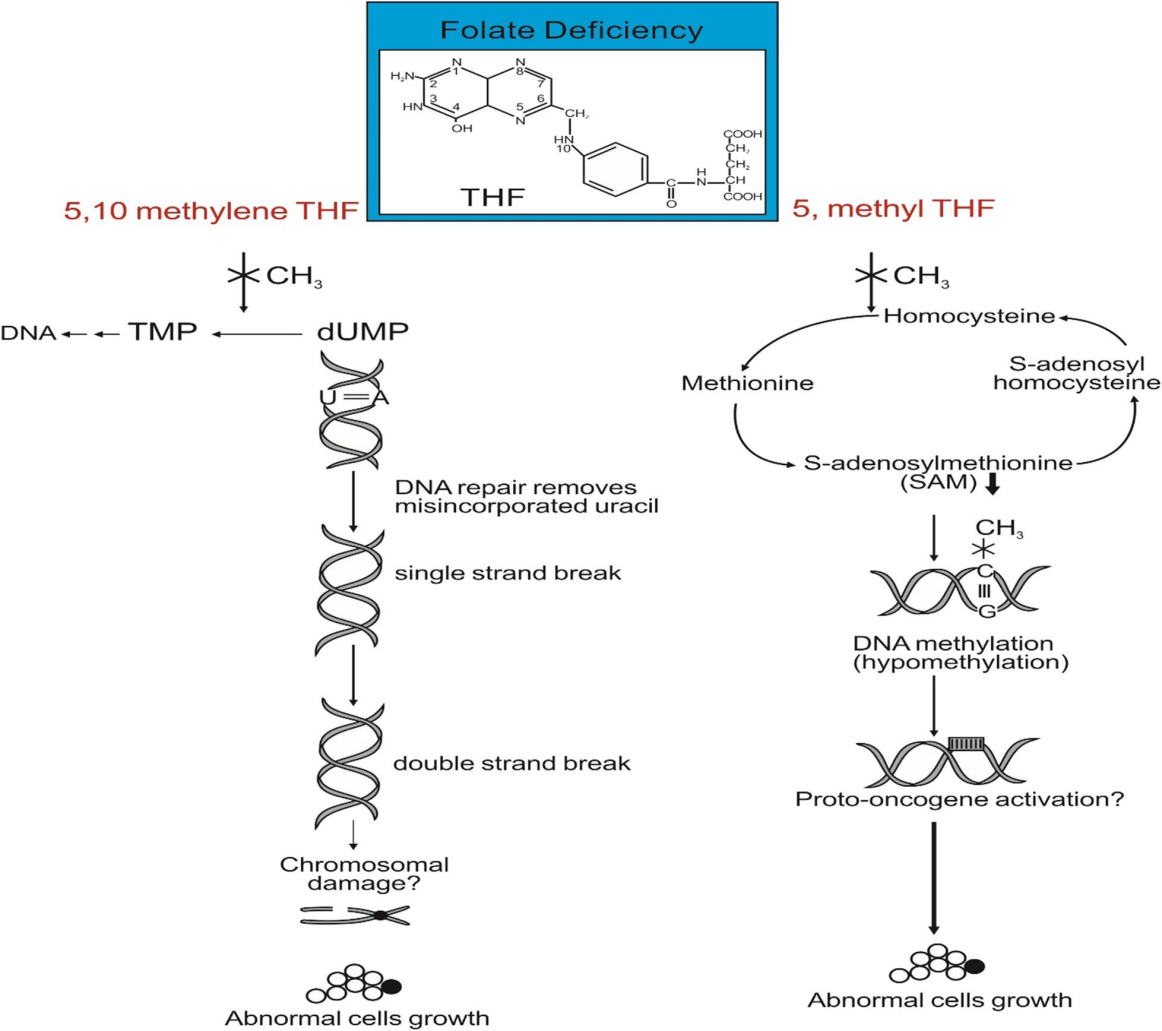
How folate deficiency leads to genomic instability? Two way: 5,10-methylene-THF, 5,10-methylenetetrahydrofolate; 5, methyl THF, 5-methylenetetrahydrofolate; THF, tetrahydrofolate; C, cytosine; G, guanine; X denotes no capability to donate a methyl group (CH_3_); TMP, thymidylate monophosphate; desoxyuridylate monophosphate, dUMP (adopted from Duthie (1999))

Folate has a dominant role in DNA metabolism, function, and repair due to its ability to methylate cytosine, thereby controlling gene expression, and its key function in nucleotide synthesis. Folate deficiency leads to genomic instability and genomic instability appears through two latent pathways (Fig. 1). The first pathway in which folate plays a significant role is altered DNA methylation. The major circulating folate type, 5-methyl-THF, acts as a cofactor in methionine synthesis (Abbasi et al. 2018). Methionine is converted to SAM methylates, the main methyl donors in DNA cytosine methylation, and methylation controls gene expression. Under folate deficiency, SAM is depleted, and methyltransferase activity is elevated, leading to DNA hypomethylation (Yi et al. 2000), insufficient proto-oncogene activation, transcription, and subsequent growth of abnormal cells or tumors (Kim et al. 1996; Fang and Xiao 2001). Proper folate concentration is important for the synthesis of DNA nucleotides, the backbone of DNA, in which desoxyuridylate monophosphate (dUMP) is changed to thymidylate monophosphate (TMP) by thymidylate synthase using 5,10-methylene-THF as a methyl donor. When folate is low, dUMP may fold and start inducing uracil misincorporation.

The second pathway by which folate deficiency modifies genomic stability position. This occurs when vitamin B_12_ is restrictively low and 5-methyl-THF cannot undergo further metabolization to yield THF. Consequently, the availability of 5,10-methylene-THF decreases, and less is available to maintain methylation reactions. However, folate, as 5,10-methylene-THF, donates a methyl group to uracil, converting it to thymine for DNA synthesis and repair. If folate concentration is limiting, uracil misincorporation increases and genomic instability develops. This condition develops as cells of the body attempt to repair themselves, breaking DNA molecules to remove uracil. When folate concentration is limited, the precursor pool of deoxynucleotide triphosphates is reduced, and misincorporation of uracil increases in “a catastrophic repair cycle” which may promote double-strand breaks, chromosomal damage, and abnormal cell growth (Fig. 1) (Reidy 1988; Blount and Ames 1994). Furthermore, a study reported that low folate may induce malignant transformation, declining SAM biosynthesis, de novo thymidine monophosphate synthesis, modified gene expression by defective cytosine methylation, or lead to the development of catastrophic cycles of aberrant DNA repair and subsequently uracil misincorporation (Ciappio and Mason 2010). Folate deficiency also promotes purine biosynthesis and increased DNA hypomethylation disorder (Kim et al. 1996; Duthie and Hawdon 1998). Specifically, when folate concentration in the body is balanced, there is reduced uracil misincorporation, inhibition of DNA excision repair, DNA stability increases, and growth of abnormal cells is limited (Duthie et al. 2000).

## Methylation cycle and the potential role of folate

**Fig. 2:**
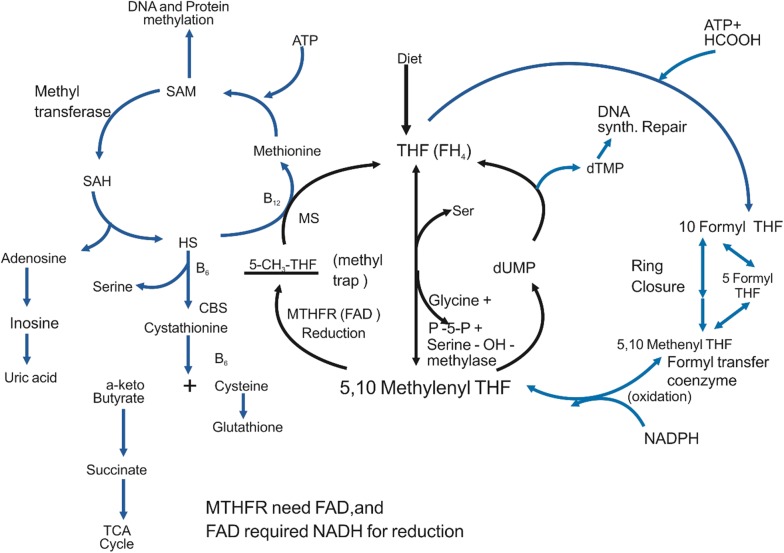
Metabolism of folate and its relationship with methionine. The key donor (SAM) is synthesized from methionine and is used to transfer a methyl group in DNA and the protein methylation cycle. Once a methyl group is transferred, it will change into *S*-adenosyl homocysteine, which further becomes homocysteine, and then methionine. Methyltetrahydrofolate (THF) and vitamin B_12_ are required as key regulatory cofactors. FAD, flavin adenine dinucleotide; NADPH, nicotinamide adenine dinucleotide phosphate

Methylation is a key reaction cycle through which a methyl group is transferred on to, enzymes, amino acids, proteins, and DNA in cells or tissue to control healing processes, cell energy, DNA expression, neurological function, liver detoxification, and immunity (Kim et al. 2009; Yang et al. 2010). Methylation occurs chemically or biologically. In chemical methylation, a methyl group is added or substituted on to a substrate. In biological methylation, the reaction is catalyzed by enzymes; this kind of methylation is mostly involved in the alteration of heavy metals, gene expression regulation, protein function, and DNA and RNA processing. Gene expression regulation mechanisms in which gene expression or function is altered without any change to the DNA sequence (e.g. DNA methylation) are the key processes underlying epigenetics. These mechanisms are essential for regular growth and related to a number of key processes including: genomic imprinting, X-chromosome inactivation, suppression of identical elements, the aging process, and carcinogenesis (Lister et al. 2009; Rana and Ankri 2016). Epigenetics is the study of phenotypic variations that arise without the underlying DNA sequence being altered (Suzuki and Bird 2008; Fazzari and Greally 2010; Colaneri et al. 2011). Epigenetic methylation processes are significantly affected by dietary levels of methyl donors, namely folate and its derivatives: THF, 10-formyl-THF, 5-formyl-THF, and 5-methyl-THF. Dietary folic acid is primarily metabolized into 5-methyl-THF after intestinal absorption. 5-Methyl-THF then passes through the liver, is reduced to dihydrofolate by dihydrofolate reductase, and then further reduced to THF before entering the folate pool. 5-Methyl-THF is the primary folate constituent carried by non-hepatic tissues, and must be polyglutamated for cellular retention and one carbon cycle coenzyme function (Rowling et al. 2002; Lucock 2007). THF is mainly produced by the action of folate polyglutamate synthetase, and secondarily produced by the conversion of 5-methyl-THF to THF through methionine metabolism. In rare situations where dihydrofolate reductase is high, folic acid is oxidized and present in the circulation system in its active form (Bailey et al. 2010). When the THF coenzyme is formed from either folic acid or dietary folate, it first changes in to 5,10-methylene-THF catalyzed by vitamin B_6_-dependent serine hydroxymethyltransferase, before being irrevocably reduced by methylenetetrahydrofolate reductase (MTHFR) into 5-methyl-THF (Fig. 2) (Scott 1999). Vitamin B_12_-dependent methionine synthase balances the fluctuation of methyl groups for remethylation of homocysteine to yield methionine and regulate *S*-adenosyl methionine synthesis. Methionine is also a methyl donor for and a cofactor in the methylation of DNA, RNA, neurotransmitters, histones, phospholipids, proteins, and other small molecules (Fig. 2) (Stover 2009). Several reactions are regulated by SAM and SAM concentration significantly affects gene transcription, genomic stability (Miranda and Jones 2007), protein localization (Winter-Vann et al. 2003), and small molecule degradation (Stead et al. 2004). DNA methylation controlling gene transcription and genetic stability is one of the most important types of reactions among the more than a hundred methylation reactions (enzymatic) mediated by SAM. However, with folate, several other dietary nutrients are required to balance one-carbon flux. For example, serine from dietary and microbial sources plays a significant role in one-carbon flux as it donates two one-carbon units as it is converted into glycine before being catabolized further (LeGrusse et al. 1993). Moreover, de novo serine synthesis using glycerophosphate as a substrate transfers a methyl group in to the one-carbon pool (Emmanuel and Kennelly 1984; Armentano 1994). Other important nutrients that play a key role in methylation reactions include: vitamin B_6_ (serine hydroxymethyltransferase activity), riboflavin (MTHFR stability), vitamin B_12_ (methionine synthase function), and choline (betaine precursor, homocysteine methyltransferase) (Combs 1998; Shin et al. 2010; Abbasi et al. 2017). If the concentration of SAM is high, MTHFR is inhibited, due to the synthesis of 5-methyl-THF and remethylation of homocysteine is reduced. Vice versa, when SAM is low, homocysteine remethylation is increased. Therefore, MTHFR activity and formation of 5-methyl-THF may mitigate the effect of the common genetic variant, 677C/T, which decreases enzymatic activity (Bailey 2009). However, S-adenosylhomocysteine (SAH) works as a strong product inhibitor of SAM-dependent methyltransferase (Hoffman et al. 1980; Finkelstein 1990). Due to the fact that hydrolysis of SAH into homocysteine is required for DNA methylation (James et al. 2002), demethylation of 5-methyl-THF does not occur if vitamin B_12_ is too low (Bassler 1997). Even if folate concentration is adequate, a shortage of vitamin B_12_ inhibits the production of methionine and SAM (Rowling et al. 2002; Reed et al. 2004). Insufficient availability of folate during cell division reduces the synthesis of thymidine, increases uracil misincorporation, and hampers DNA repair. Insufficient folate can decrease the capacity of cells to restore DNA under oxidative or alkylation conditions (Duthie et al. 2000) and therefore, impedes cell proliferation. This mutagenic condition may have negative effects including a higher frequency of chromosomal breaks and abnormal cell growth (Lamprecht and Lipkin 2003).

## Folates and vitamin B_12_ interaction

Vitamin B_12_ plays an important and significant role in the metabolism of homocysteine and methionine; B_12_ and folate promote remethylation of homocysteine which in turn allows for methionine synthesis. However, homocysteine is also converted into cysteine (a precursor of glutathione) through actions of cystathionine-β-synthase and cystathionase enzymes. Ruminal bacteria of mature animals have the ability to synthesize B vitamins using folic acid and B_12_ vitamin (Lardinois et al. 1944; NRC 2001) for their use and use by the host organism. B vitamins production is important, because B vitamins act as cofactors or coenzymes for the breakdown and absorption of fatty acids, proteins, non-structural or structural carbohydrates, and genomic material (Combs 2012). It has been noted in many studies that B vitamins supplements can promote methionine synthesis, the remethylation cycle, milk production and improved composition, and metabolic efficiency of high yielding ruminants (Shaver and Bal 2000; Graulet et al. 2007). All B-vitamins, folate, and folic acid (pteroyl-l-glutamic acid) are beneficial nutrients for optimum productivity in dairy animals, but vitamin B_12_ is an especially key agent in folate metabolism. Although vitamin B_12_ is synthesized by rumen bacteria and archaea, synthesis is also dependent on cobalt concentration (Martens et al. 2002; Abbasi et al. 2013). Furthermore, two major vitamin B_12_-dependent enzymes are present in dairy animals, methionine synthase and second methylmalonyl-CoA mutase. Methionine synthase transfers a methyl group from 5-methyl-THF to homocysteine to yield methionine and THF. However, methylmalonyl-CoA mutase transforms methylmalonyl-CoA into succinyl-CoA for utilization in the Krebs cycle reaction and then, the gluconeogenesis process. A study found vitamin B_12_ concentrations in dairy cows serum to be 2.4, 2.0 and 1.2 ng/ml at 21, 7 and 120 days after parturition (McDowell 2000). Vitamin B_12_ level declined from 21 to 7 days prepartum (Kincaid et al. 2003). Furthermore, another study using multiparous cows, presented that vitamin B_12_ concentration significantly decreased in serum from 5.7 ng/ml at 55 days prepartum to 2.3, 2.0 and 1.9 ng/ml at 20, 7 and 120 days after parturition respectively (Kincaid and Socha 2007). Synthesis of B_12_ and folic acid in the rumen of dairy cattle has been measured to be 73.0–79.8 and 16.5–21.0 mg/day respectively (Santschi et al. 2005; Schwab et al. 2006), and these rates of synthesis significantly affect the amount of vitamins secreted into milk (Ferlay et al. 2013). Some studies reported that vitamin B_12_ deficiency is sometimes mitigated by high folate concentration (Selhub et al. 2009), and that vitamin B_12_ secretion into the milk can be managed in dairy animals through dietary supplementation of vitamin B_12_. It has been recorded that milk contains highly variable concentrations of vitamin B_12_ among different farms, with the normal range fluctuating from 2.2 to 3.9 ng/ml (Duplessis 2014). In studies that supplemented diets with folic acid, folic acid utilization decreased in cows tissues in early lactation due to vitamin B_12_ deficiency and because folic acid was trapped in serum in its methylated form, which significantly inhibits demethylation. Current knowledge suggests that methionine synthesis is dependent on vitamin B_12_ enabling the conversion of extracellular 5-methyl-THF into polyglutamate THF, which is utilized in nucleotide synthesis, and that therefore, folate and vitamin B_12_ levels should be managed in rations (Sirotnak and Tolner 1999; Chassaing et al. 2011). Moreover, during catabolism of homocysteine, serum folate concentration was noted to be higher with the production of cysteine, and vitamin B_12_ concentration was low. However, methionine and serum clearance of folate was improved in multiparous cattles when fed folic acid with rumen-protected methionine supplementation, suggesting that vitamin B_12_ synthesis and supply was inadequate and inhibited folate production (Girard et al. 2005). Thus, vitamin B_12_ may be a limiting factor for folic acid metabolism in dairy animals (Girard and Matte 1998).

## The folic acid requirement, supplementation, and performance of dairy ruminants

During lactation and gestation in high-producing dairy animals, methylneogenesis, DNA synthesis, cell division, epigenetic processes, and others important processes deeply depend on folate metabolism. While a supply of nucleic acids from microbial digestion might reduce the burden on the DNA cycle, gluconeogenesis and methylneogenesis is high in high-producing dairy animals, particularly during early lactation (Abbasi et al. 2018). However, in some cases, precursors of de novo synthesis of methylated compounds were found to be insufficient. In these cases, folate supplementation would improve the transport of one-carbon units. Consequently, a supply of both co-factors (folate and B_12_) and methyl donors were essential for maximum dairy performance. Some studies reported that, in growing steers, duodenum folate concentrations were marginal, and non-gestating cattle had serum folate concentrations greater than that of gestating cattle (Arbeiter and Winding 1973; Tremblay et al. 1991). In dairy cows, total serum folate level was noted to decline by about 40% within the 2 months prior to a calving (Girard et al. 1989). Changes in serum folate concentration provide signs as to the relationship between folate supply and folate utilization in tissues and the change in that relationship during different physiological stages. Folic acid supplementation increased the placental and colostral transmission of folate to the calf. Other effects on blood hemoglobin, growth, birth weight, and feed intake of the mother were not found to be significant during the 10 weeks prior to birth. Other studies reported that folic acid injection promotes a significant increase in milk protein content in multiparous cows, but has no significant effect in primiparous cows (Girard et al. 1995; Girard and Matte 1998). Folate and vitamin B_12_ concentrations in plasma increased during dietary supplementation of both nutrients (Girard and Matte 2005). However, during dietary supplementation of cobalt, vitamin B_12_ serum level during early lactation was lower in primiparous cattles than in multiparous cattles (Girard and Matte 1999). This study also reported that in early lactation in dairy cows, serum vitamin B_12_ concentration was lower, but serum folate concentration peaked, particularly during folic acid dietary supplementation. However, the situation was reversed 8–12 weeks after lactation, when serum vitamin B_12_ was higher than serum folate in supplemented cows (Girard and Matte 1999). Previous studies reported that folic acid is also synthesized by ruminal micro-organisms, but folic acid from ruminal-synthesis is about 16.5–21.0 mg/day (Santschi et al. 2005), less than the folic acid requirement estimated recommended for dairy cows by the National Research Council (35 mg/day) (NRC 2001). Furthermore, ruminal microorganisms degrade around > 0·95 of supplemented folic acid in dairy cows rations (Santschi et al. 2005). Thus, rumen-protected folic acid (RPFA) is necessary for high-producing ruminants.

Folic acid is required by ruminal microorganisms (Girard et al. 1994; Wejdemar 1996), and dietary supplementation of folic acid has been shown to increase cellulolytic bacterial population (Wejdemar 1996), cellulose digestion (Ragaller et al. 2010), ruminal fermentation (Hayes et al. 1966), ammonia nitrogen (NH_3_-N) utilization (Wejdemar 1996), and concentration of milk yield, or milk protein (Girard et al. 1995; Girard and Matte 1998). However, other studies found that ruminal fermentation (Chiquette et al. 1993; Girard et al. 2009) and NH_3_-N utilization (Girard et al. 1994; Ragaller et al. 2010) were not changed by supplementary folic acid. Another study reported that with increasing dietary crude protein (CP) and RPFA supplementation, ruminal total volatile fatty acid (VFA) concentration in dairy animals was consistent with degradability, bacterial population size, and microbial enzymatic action (Broderick 2003; Wang et al. 2016). Ruminal pH was lower in steers under RFPA supplementation, and this was attributed to an increased ruminal total VFA concentration, urinary total purine derivative excretion, and ruminal NH_3_-N utilization for microbial protein synthesis (Froetschel et al. 1989; Wang et al. 2016; Kolver and Deveth 2002). Dietary supplementation of both CP and RPFA promoted microbial growth, increased microbial enzyme activity, increased in situ ruminal digestibility, and improved total VFA production in beef cattles (Wang et al. 2017). Supplementation of vitamin B_9_ and B_12_ improved the condition and performance of high-producing dairy cows, especially through the critical period around calving and early lactation (Preynat et al. 2009). Dietary supplementation of vitamins B_9_ and B_12_ together increased production of milk components and milk yield in dairy cows (Ouattara et al. 2016). Metabolic efficiency, dry matter intake, and milk performance were increased with supplementation of folic acid in dairy cattles, but plasma glucose and hepatic lipids decreased when dairly cattles were fed vitamin B_12_ together with folic acid (Graulet et al. 2016). Folic acid and B_12_ vitamin supplementation resulted in an approximately 12% increase in milk yield in multiparous cows between 3 weeks before calving and 16 weeks of lactation (Preynat et al. 2010). Some studies found that populations of *R. albus*, *R. flavefaciens*, *B. fibrisolvens*, and *F. succinogene* and the activity of cellobiase, xylanase, pectinase, and α-amylase increased quadratically with increasing PRFA supplementation. Furthermore, overall fiber degradability increased by 42% and in vitro and in situ ruminal acid detergent fiber degradability increased when rations were supplemented with folic acid (Wang et al. 2016). Studies on dairy cows reported a supplemented folic acid dose of 2 mg/kg BW (body weight) (Girard et al. 1994), 3–6 mg/kg BW (Girard et al. 2009), approximately 1.65 mg/kg BW (Ragaller et al. 2010), and 0.2 mg/kg BW (Wang et al. 2017).

In conclusion, folic acid (Folate) supplementation offers a therapeutic for hematological, immunological, oxidative, and genomic complications, increases 5-methyltetrahydrofolate levels, and promotes the synthesis of milk protein from dietary protein in ruminants. It is imperative to adjust ruminants folate requirements precisely and according to the stage and physiological period. More the study is needed concerning whether supplementation of folate in rations should be rumen-protected or rumen-unprotected. More research is also necessary for developing nutritional policies that promote vitamin synthesis by ruminal microflora and for finding the balance between supply and demand of folic acid in ruminants rations.

## Abbreviations

DNMTs: DNA methyltransferases
dUMP: desoxyuridylate monophosphate
Fra: folate receptor alpha
Frb: folate receptor beta
MTHFR: methylenetetrahydrofolate reductase
MTRR: methylenetetrahydrofolate
PCFT: proton coupled folate transporter
RFC: reduced folate carrier
SAM: *S*-adenosylmethionine
THF: tetrahydrofolate
TMP: thymidylate monophosphate
